# Electrochemical Impedance Immunoassay for ALS-Associated Neurofilament Protein: Matrix Effect on the Immunoplatform

**DOI:** 10.3390/bios13020247

**Published:** 2023-02-09

**Authors:** Omair Adil, Mohtashim H. Shamsi

**Affiliations:** School of Chemical and Biomolecular Sciences, Southern Illinois University, 1245 Lincoln Drive, Carbondale, IL 62918, USA

**Keywords:** amyotrophic lateral sclerosis, neurofilament-light, electrochemical impedance spectroscopy, immunoassay, diagnostics

## Abstract

Amyotrophic Lateral Sclerosis (ALS) is a neurodegenerative disorder, which has complex diagnostic steps. Electrochemical immunoassays may make the diagnosis simpler and faster. Here, we present the detection of ALS-associated neurofilament light chain (Nf-L) protein through an electrochemical impedance immunoassay on reduced graphene oxide (rGO) screen-printed electrodes. The immunoassay was developed in two different media, i.e., buffer and human serum, to compare the effect of the media on their figures of merit and calibration models. The label-free charge transfer resistance (R_CT_) of the immunoplatform was used as a signal response to develop the calibration models. We found that exposure of the biorecognition layer to human serum improved the impedance response of the biorecognition element with significantly lower relative error. Moreover, the calibration model obtained in the human serum environment has higher sensitivity and a better limit of detection (0.087 ng/mL) than the buffer medium (0.39 ng/mL). The analyses of the ALS patient samples show that concentrations obtained from the buffer-based regression model was higher than the serum-based model. However, a high Pearson correlation (r = 1.00) between the media suggests that concentration in one medium may be useful to predict the concentration in the other medium. Moreover, the Nf-L concentration appears to increase with age in both male and female groups, while overall higher Nf-L was found in the male group than the female group.

## 1. Introduction

Amyotrophic Lateral Sclerosis (ALS) is a fatal neuromuscular disease that causes death within five years of onset of symptoms [1]. Diagnosis for ALS relies on a detailed history of the symptoms, a series of muscle testing to rule out other diseases, and imaging tests [2]. The availability of a range of sensitive tools to diagnose the disease is difficult in a resource-limited region [3]. However, recent progress in the identification of ALS-associated biomarkers present in biological fluids is promising for developing sensitive and inexpensive electroanalytical diagnostic protocols [4,5,6,7]. In contrast to current ALS diagnostics methods, electrochemical detection methods have emerged as a promising alternative due to their simplicity, quick process, affordability, and sensitivity [8,9]. These methods can be integrated into miniaturized platforms, such as screen-printed electrodes and microfluidic chips [10,11,12], to detect a wide range of analytes in different biological samples, including blood [13], urine [14], sweat [15], and saliva [16]. The electrochemical detection platform incorporates a diverse range of surfaces, harnessing the unique properties of nanomaterials to enhance its sensing capability [17,18]. One such material is reduced graphene oxide (rGO), a class of two-dimensional nanomaterial, which is utilized for its convenient functionalization in immobilizing biorecognition elements [19]. rGO material shows remarkable electrochemical conductivity, high surface area, biocompatibility, and hydrophilic characteristics, resulting in improved sensitivity and detection limits in the electrochemical detection process [20].

In past 25 years [21], a number of protein biomarkers have been linked to ALS such as Polydipeptide Repeats (DPRs), Neurofilament Heavy Chain (Nf-H), Neurofilament Light Chain (Nf-L), and Phosphorylated Neurofilament Heavy (pNfH) and Light Chain (pNfL) [22]. Currently, these biomarkers were detected in cerebrospinal fluid (CSF) due to their high concentration in the biological fluid [23,24], while obtaining a CSF sample is highly invasive. Recently, Nf-L received significant attention as a biomarker for early-stage diagnosis [25], which can be detected in serum [25,26]. We expect that the detection of Nf-L in serum using a rapid, label-free, and sensitive detection method will make the diagnosis procedure less invasive, thus less painful, for ALS patients.

**Scheme 1:**
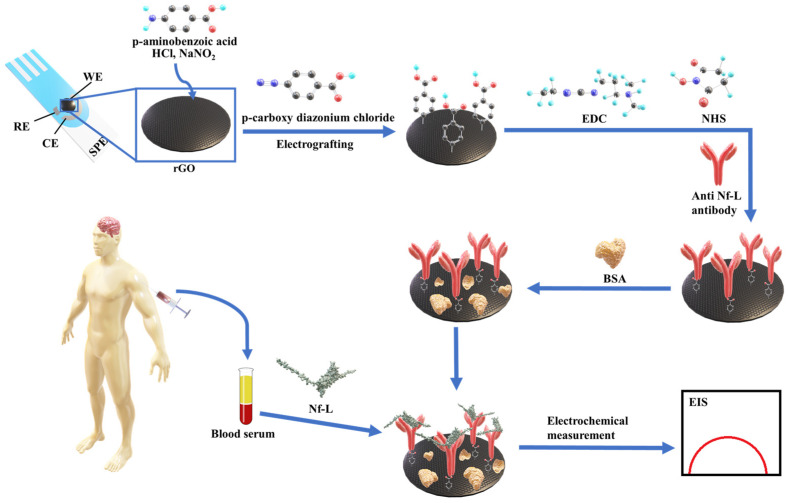
Depicting step by step preparation of the electrochemical immunosensing platform on rGO screen-printed electrodes and the detection of ALS-associated Nf-L biomarker in patients sera using the developed immunosensing platform.

The detection of diagnostic biomarkers in a complex biological medium (e.g., serum) usually requires tedious sample preparation to enrich the biological sample with a desired biomarker. In contrast, testing enriched and complex target samples via highly sensitive techniques, such as impedance, may fall beyond the dynamic range of the calibration model. Therefore, dilution of such samples might be necessary but also at risk of compromising accuracy and precision. In this work, we have detected the ALS-associated Nf-L protein in patients sera using impedance-based electrochemical immunoassay. Scheme 1 depicts the detection strategy where an antibody biorecognition layer was formed on a reduced graphene oxide (rGO) screen-printed electrode through an electrografting method where an anti-Nf-L antibody is immobilized on the rGO surface through a 4-carboxyphenyl linker [27,28]. Then, Nf-L was detected using the immunoplatform by monitoring the label-free impedance signal of the detection platform. The immunoassay was employed in buffer and serum to compare the effects of the media on the figures of merit and regression model of the assay and to observe the effect on the analysis of real samples. This detection strategy is simple, label-free, and less invasive compared to other electrochemical methods that used brain tissues and various signal amplification methods [29,30,31].

## 2. Experimental

### 2.1. Materials

Synthetic neurofilament light chain (Nf-L)—a human recombinant protein (P01)—with theoretical molecular weight 87.9 kDa, was purchased from Abnova corporation, Taipei, Taiwan. Anti-Nf-L monoclonal antibody (DA2) was procured from Invitrogen, Thermo Fisher Scientific, Rockford, IL, USA. Commercial human serum (from human male AB plasma, USA origin, sterile-filtered) was purchased from Saint Louis, MO, USA. Bovine serum albumin (BSA) lyophilized powder (≥96%), sodium nitrite (≥99.0%) and 4-aminobenzoic acid (≥99%) were purchased from Millipore-Sigma, Milwaukee, WI, USA. ALS patient serum samples were acquired from National ALS Biorepository (Center for disease control and prevention, Atlanta, GA, USA), obtained, and stored at −80 °C. Additionally, 1-ethyl-3-(3-dimethylaminopropyl)carbodiimide (EDC) hydrochloride, *N*-hydroxysuccinimide (NHS) and [2-(N-morpholino)ethanesulfonic acid] (MES) 0.1 M, with 0.9% sodium chloride were purchased from ThermoFisher Scientific, Rockford, IL, USA. Potassium ferricyanide (99+%) and potassium ferrocyanide trihydrate (99+%) were purchased from Acros Organic, New Jersey, NJ, USA, while phosphate buffer saline 10× were obtained from Sigma life science, Milwaukee, WI, USA. All the solutions were prepared in ultrapure water (18.2 MΩ·cm) from Barnstead Smart2Pure 3LPH, Thermo Scientific, Asheville, NC, USA. Reduced graphene oxide modified screen printed carbon electrode (DRP-110RGPHOX) were purchased from Metrohm, Riverview, FL, USA. All electrochemical measurements were performed employing Metrohm Autolab PGSTAT204 FRA32M (Riverview, FL, USA) electrochemical workstation connected with Metrohm Dropsens adapter (DRP-DSC4MM70734) inside Metrohm Autolab Faraday cage Riverview, FL, USA.

### 2.2. Preparation of Immunoplatform

A biorecognition layer was prepared on the reduced graphene oxide (rGO) screen-printed electrode (SPE). The SPE comprised of an rGO working electrode with 4 mm diameter, a carbon counter electrode, and a silver pseudoreference electrode. The rGO working electrode was first modified with carboxyphenyl group using electrografting method [27,28]. First, 2 mM of NaNO_2_ was added into 2 mM of 4-aminobezoic acid prepared in 0.5 M HCl to generate diazonium cation. After 5 min of reaction, 150 μL of the solution was dropped on to the rGO surface. Covalent immobilization of 4-carboxyphenyl group was obtained by electrografting using cyclic voltammmtery (CV) from 0.4 to −0.6 V for 3 cycles at 200 mV/s scan rate. For anti-Nf-L antibody immobilization, 10 μL of 100 mM EDC and 20 mM NHS prepared in 100 mM MES buffer (pH 5) was dropped on carboxy phenyl modified rGO surface and the electrode was incubated around 8 °C for 1 h. The surface was carefully washed with MES buffer followed by incubation of 10 μL of 20 ng/mL anti-Nf-L antibodies, prepared in 1× PBS buffer (pH 7.4) for 3 h at 8 °C. Then, the surface was washed with PBS buffer and exposed to 10 μL of 1% BSA as a blocking layer for 1 h followed by washing with PBS buffer. The biolayer was also exposed to 10 μL undiluted serum for 15 min followed by washing with PBS buffer. Synthetic Nf-L targets in the concentration range 0.01 to 1.5 ng/mL were prepared in 1× PBS and in serum separately and were immobilized in respective biorecognition layer for 15 min followed by washing with PBS buffer.

### 2.3. Nf-L Detection by EIS

Each of the biolayer preparation steps and target immobilization steps were electrochemically characterized to confirm the modification. EIS was performed using 1 mM K_4_[Fe(CN)_6_]/K_3_[Fe(CN)_6_] (1:1) prepared in 1× PBS (pH 7.4) as a soluble redox active probe. The EIS measurements were performed in frequency range of 100 kHz to 0.150 Hz with an applied DC potential (open circuit potential), and AC pulse of 5 mV amplitude. For simulations, Z-view version 3.5 h was used to fit the EIS data into a modified Randles equivalent circuits. The experimental and extracted values of fitting elements were normalized by electroactive area at each step. For real samples, serum samples from ALS patients were obtained National ALS biorepository and stored in −80 °C freezer. Aliquots of samples were taken out in small Eppendorf tube and diluted with 1× PBS (pH 7.4) and then around 150 μL of sample was incubated for 15 min in closed container with humid environment at room temperature. The electrodes were then washed with 10 μL of 1× PBS for three times before EIS measurements.

## 3. Results and Discussion

### 3.1. Preparation of Immunoplatform

**Figure 1:**
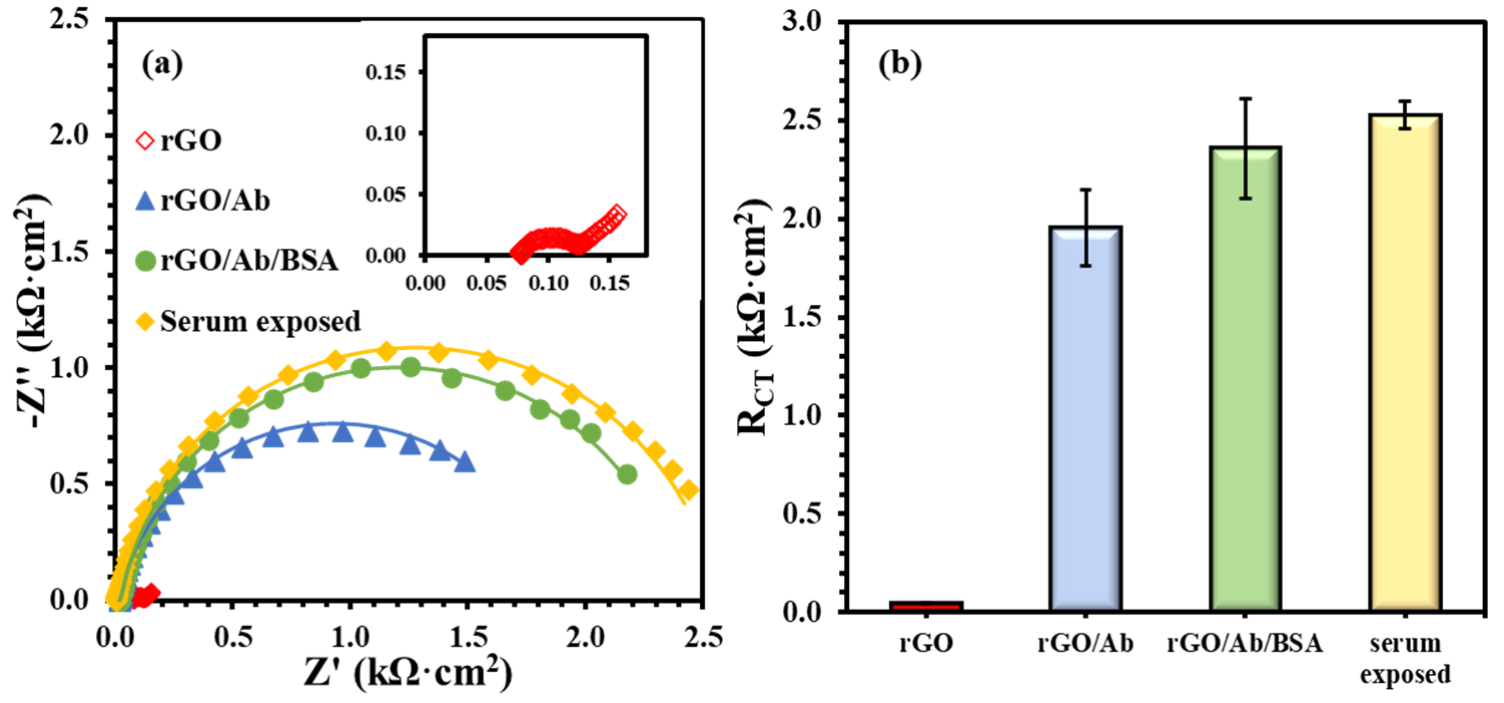
(**a**) EIS plots for biolayer formation including unmodified rGO surface (inset), anti-Nf-L antibody rGO/Ab, after 1% BSA treatment rGO/Ab/BSA, and after the human serum exposure. (**b**) Comparing R_CT_ values after modifying the rGO electrode with each successive layer. The error bars represent n ≥ 4 distinct replicates.

The immunoplatform on rGO screen-printed electrode was prepared by immobilizing anti-Nf-L antibody on the electrodes using the electrografting method [27,28]. Figure 1 presents the formation of the biorecognition layer comprising bare rGO, antibody (anti-Nf-L) onto rGO, and BSA treatment of the immobilized antibody. Figure 1a shows the Nyquist form of the EIS plots of main layers, i.e., electrode (rGO), antibody (G/Ab), complete biolayer (rGO/Ab/BSA), and human serum exposed biolayer. Figure 1b summarizes the impedance of each layer, i.e., charge transfer resistance (R_CT_), emerged from steric hindrance to the soluble redox probe. The unmodified rGO surface has extremely low resistance (0.040 ± 0.003 kΩ·cm^2^), which increased almost two orders of magnitude (1.95 ± 0.20 kΩ·cm^2^) after covalent immobilization of the anti-Nf-L on the electrografted rGO surface. After the exposure of rGO/Ab to 1% BSA, the resulting rGO/Ab/BSA biolayer had an impedance of 2.4 ± 0.25 kΩ·cm^2^ with RSD ~ 10%. The purpose of BSA in preparing such platforms is to reduce non-specific adsorption. However, the 10% relative error indicates that the platform is prone to a high rate of false-positive signals. Then, we exposed the rGO/Ab/BSA biolayer to an undiluted commercial human serum followed by washing with the buffer. The exposure to the human serum significantly improved the precision of the biolayer response, i.e., 2.5 ± 0.07 kΩ·cm^2^ and RSD ~3%. Rationally, human serum is more biologically relevant toward biorecognition layer and the serum exposure may have covered some exposed rGO surface left after the adsorption of BSA [32].

### 3.2. Nf-L Detection in Buffer and Serum Media

**Figure 2:**
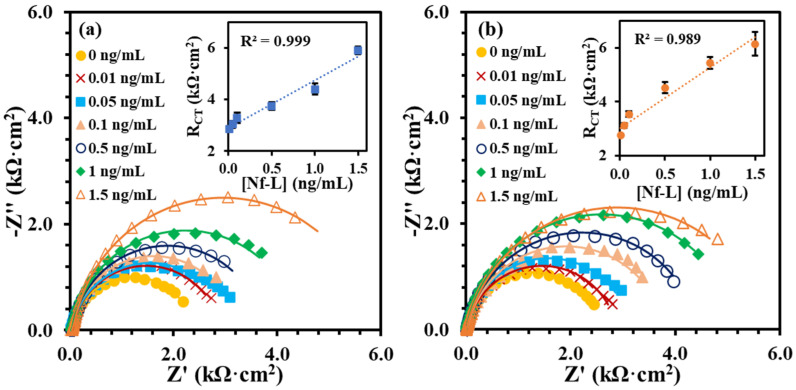
EIS signals of the synthetic Nf-L target captured at the immunoplatform from concentration range (0.01 to 1.5 ng/mL) in buffer medium PBS pH 7.4 (**a**) and in serum (**b**). The insets in (**a**,**b**) show corresponding calibration plots of R_CT_ vs. concentration. Each error bar represents n ≥ 3 replicates.

**Figure 3:**
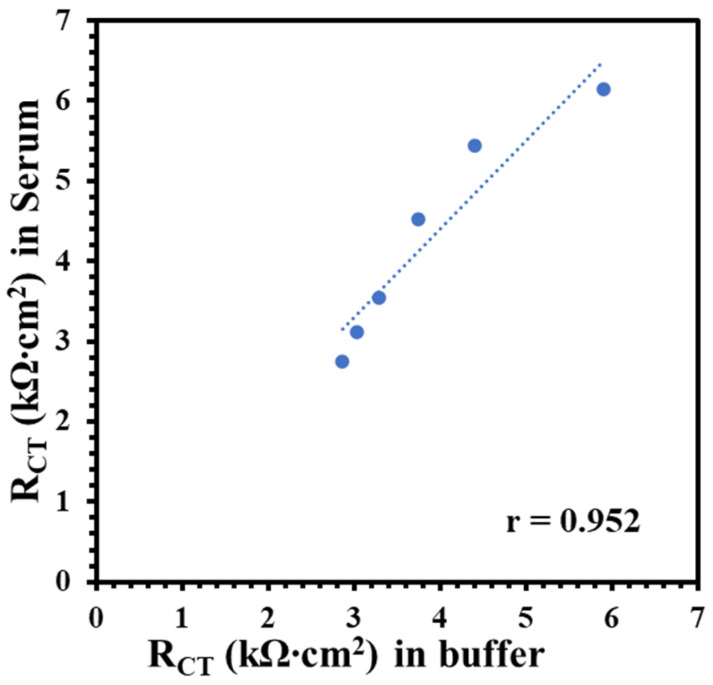
Correlation plot between R_CT_ of synthetic Nf-L targets in commercial human serum with the R_CT_ obtained in 1× PBS buffer. Pearson correlation coefficient r = 0.952 shows high correlation between target signals obtained in these two media.

The Nf-L concentration in human serum has been reported in the range 0.05–0.879 ng/mL for early-stage ALS and 0.02–4.2 ng/mL for late-stage ALS patients [33,34]. Despite the wide and varied ranges of Nf-L concentration in human serum, the median values for both stages are very similar, i.e., 0.26 ng/mL and 0.2 ng/mL, respectively. Clinical serum has a complex matrix, which makes it highly challenging to detect the target biomarker. Protein enrichment is needed for the detection of low abundant target in serum. However, sample preparation may result in quantitative loss of molecule of interest [35]. It is critical to know that buffer-based calibration model usually do not relate with real sample matrix, such as serum samples. To better understand the matrix effect on the immunoplatform, it is important to compare a buffer-based calibration with a serum-based calibration. It was assumed that an appropriate regression model may be around the reported median value of the Nf-L in serum. Therefore, we prepared and analyzed the synthetic Nf-L target in concentration range of 0.01–1.5 ng/mL prepared in PBS buffer and filtered human serum. Figure 2a shows EIS signals of the synthetic Nf-L target in PBS buffer. The increase in R_CT_ signal with the concentration confirms the immobilization of the target on the immunoplatform. The dynamic response was observed in the concentration range that can be observed in the calibration curve shown in inset of Figure 2a. The limit of detection (LOD) was calculated as 0.39 ng/mL (S/N = 3) from linear regression equation (*y* = 1.9*x* + 2.9) obtained in the buffer medium. Figure 2b represents EIS response of the synthetic Nf-L protein prepared in human serum along with the regression curve in the inset. The response of the concentrations were higher in the serum medium, which was expected due to the complexity of the medium. Nevertheless, the sensitivity (slope) of the curve was higher (*y* = 2.4*x* + 3.0) and LOD was calculated as 0.087 ng/mL (S/N = 3), which is one order of magnitude better than that of the buffer medium. We assume that the better precision of the serum-exposed biolayer improved the LOD from serum-based calibration plot due to similar matrix. We performed comparative analysis of R_CT_ signals of two data sets obtained from serum and buffer calibration media and found no significant difference in variance (*p* > 0.08) and in means (*p* > 0.6). This further verifies that the results obtained from both media are correlated. The correlation between the R_CT_ signals obtained from buffer and serum-based calibration curve (Figure 3) shows that both calibration models are reasonably correlated as indicated by the Pearson correlation coefficient r = 0.952. Of note, we used monoclonal anti-Nf-L antibody, which is very specific to its target antigen. Therefore, we expected no significant nonspecific interference, which was verified by the high correlation between the buffer and serum matrix results. Due to the correlation, we used both regression models to calculate concentrations of Nf-L in the diluted sera samples from ALS patients.

### 3.3. Target Nf-L Determination in ALS Patients’ Serum

**Figure 4:**
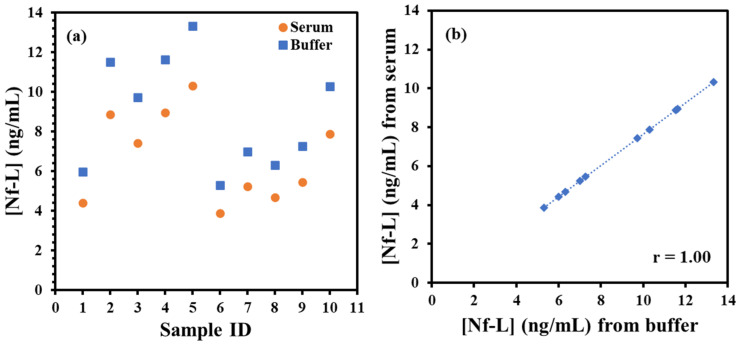
(**a**) Plot of target Nf-L concentration in ALS serum samples. The concentrations calculated from serum-based calibration plot (orange circle) and buffer-based calibration plot (blue square). (**b**) shows correlation plot between the concentration of Nf-L in real ALS samples calculated from serum-based calibration model versus buffer-based calibration model. Pearson correlation coefficient r = 1.00 shows very high correlation between these two media.

Finally, ALS sera samples were tested from male and female groups of varied ages to determine Nf-L concentration using the immunoplatform and the regression models. The samples were 10× diluted in 1× PBS buffer (pH 7.4) followed by exposure to immunosensing platform. After the brief exposure, the EIS measurements were performed preceded by washing as described above. Table 1 shows the R_CT_ signals of the sera samples normalized by the electroactive area of the electrodes and corresponding concentration of Nf-L calculated from buffer- and serum-based regression equations. The concentrations obtained were multiplied with 10× dilution factor to estimate the actual concentration in the sera. The serum dilution may be useful when target concentration in patient is expected to present at elevated level than the dynamic range of the regression model such as in case of late-stage ALS (4.2 ng/mL) [36]. The concentrations of the Nf-L from patients were plotted in male and female groups in Figure 4a. Interestingly, we found that Nf-L concentration tends to increase with age in both male and female patients. It is important to note that there have been inconsistent reports on the correlation between Nf-L concentration and age of ALS patients, for instance, a report states no direct correlation [37], while another report shows Nf-L concentration correlation with the age of healthy controls and other neurological diseases [38]. We also observed average Nf-L concentration higher in the male group than the female group, which is also not proven from previous studies. Since the work of the biomarker identification and their estimation in biological fluids is still in progress, therefore, more reports are expected in the future for conclusive evidences. Figure 4b shows the high correlation (r = 1.00) between the concentrations obtained from buffer-based calibration and serum-based calibration models, which indicates that the correlation can be used as predictive model to find the concentration in one medium from other medium and vice versa.

There have been several recent studies on electrochemical detection of Nf-L. However, these studies relied on complex biorecognition elements and label for signal amplification. One of such detection strategies involved sandwich type immunoassay complexes formed over magnetic microbead, which was conjugated with horse radish peroxidase (HRP) to generate electroactive product for amperometric detection [31], In another work, electrochemical detection was based on ratio of glycosylated Nf-L (oNf-L) to total Nf-L (tNf-L) was determined [29]. Both targets were detected in buffer diluted serum where tNf-L detection involved sandwich type immunoassay bearing nanoparticles over gold electrode surface. Nanoparticles containing Cu^2+^ provides electrochemical reduction signal to quantify tNf-L. While, oNf-L detection involved horseradish peroxidase–wheat germ agglutinin (WGA–HRP) complex–specifically immobilized on oNf-L, which then catalyzed reduction of O_2_ from H_2_O_2_. Nf-L was also detected on metal organic framework-derived material (ZrO_2_@La_2_O_3_) using a simple immunoassay for voltametric detection [30]. Nevertheless, such metal organic frameworks are expensive materials compared to graphene oxide which is simple to synthesize and modify through chemical and electrochemical methods as we described in this report. In our work, using the serum-based calibration we were able to achieve LOD as low as 0.087 ng/mL, which is sensitive enough to detect reported mean concentration of Nf-L in serum samples of neurodegenerative disease patients (0.65 ng/mL) [39].

## 4. Conclusions

In this work, we demonstrated the effect of immunoassay medium on electrochemical detection of ALS-associated Nf-L protein biomarker. The immunoplatform was prepared on reduced graphene oxide screen-printed electrodes by immobilizing the anti-Nf-L antibody through the electrografting method. The final biorecognition layer showed improved charge transfer resistance response when exposed to human serum and the relative standard error reduced from 10% to 3%. The regression models obtained in the serum medium showed higher sensitivity and better limit of detection (0.087 ng/mL) than the buffer medium (0.39 ng/mL). Both calibration models were found reasonably correlated (r = 0.952) and used to estimate Nf-L concentration in patient serum samples. The estimation of Nf-L from buffer-based regression was found slightly higher than the serum-based regression model but the concentrations were found highly correlated (r = 1.00), which can be used to predict a concentration in serum medium using the concentration from buffer medium and vice versa. Interestingly, we found increase in Nf-L level with age in both male and female groups of ALS patients. We also found an average higher concentrations in the male group than the female group. The immunoplatform developed here is label-free, simple, and sensitive to detect Nf-L for ALS diagnosis through a less-invasive method.

## Figures and Tables

**Table 1:** Charge transfer resistance (R_CT_ signals) of ALS serum samples and the concentration of Nf-L obtained in serum-based and PBS buffer-based calibration model. The data is divided into two groups based on gender and it represents varied ages. Actual concentration of Nf-L in the serum was calculated by multiplying the concertation obtained from the regression model with 10× dilution factor. R_CT_ and concentration values of each sample show standard error for n ≥ 3 replicates.

Sample ID	Gender	Age	R_CT_ (kΩ∙cm^2^)	Nf-L (ng/mL) Serum	Nf-L (ng/mL) Buffer
1	Male	40 s	4.1 ± 0.2	4.4 ± 0.7	6.0 ± 0.9
2		50 s	5.1 ± 0.4	8.9 ± 1.6	12 ± 2.0
3		60 s	4.8 ± 0.5	7.4 ± 2.2	9.7 ± 2.7
4		70 s	5.1 ± 0.4	8.9 ± 1.8	12 ± 2.3
5		80 s	5.5 ± 0.3	10.3 ± 1.3	13 ± 1.6
6	Female	30 s	3.9 ± 0.2	3.9 ± 1.0	5.3 ± 1.2
7		50 s	4.2 ± 0.3	5.2 ± 1.1	7.0 ± 1.4
8		60 s	4.1 ± 0.2	4.7 ± 0.8	6.3 ± 0.9
9		60 s	4.3 ± 0.4	5.5 ± 1.8	7.3 ± 2.2
10		70 s	4.9 ± 0.2	7.9 ± 0.8	10 ± 1.0

## Data Availability

Not applicable.
